# Correction: Dallons et al. GPR91 Receptor Mediates Protection against Doxorubicin-Induced Cardiotoxicity without Altering Its Anticancer Efficacy. An In Vitro Study on H9C2 Cardiomyoblasts and Breast Cancer-Derived MCF-7 Cells. *Cells* 2020, *9*, 2177

**DOI:** 10.3390/cells14201574

**Published:** 2025-10-10

**Authors:** Matthieu Dallons, Esma Alpan, Corentin Schepkens, Vanessa Tagliatti, Jean-Marie Colet

**Affiliations:** Department of Human Biology & Toxicology, Faculty of Medicine and Pharmacy, University of Mons, Place du Parc 20, 7000 Mons, Belgium; matthieu.dallons@umons.ac.be (M.D.); esma.alpan@student.umons.ac.be (E.A.); corentin.schepkens@umons.ac.be (C.S.); vanessa.tagliatti@umons.ac.be (V.T.)

## Error in Figure 1

In the original publication [1], there was a mistake in Figure 1. Indirect immunofluorescence staining of the GPR91 receptor on H9C2 and MCF-7 cells. The wrong picture for the “secondary AB + DAPI” condition for H9C2 cells was used. The picture used was the same as the “primary AB + secondary AB + DAPI” condition for H9C2 cells, with a different frame and with the blue channel reading only. The corrected figure, Figure 1. Indirect immunofluorescence staining of the GPR91 receptor on H9C2 and MCF-7 cells, appears below.

The authors state that the scientific conclusions are unaffected. This correction was approved by the Academic Editor. The original publication has also been updated.

## Figures and Tables

**Figure 1:**
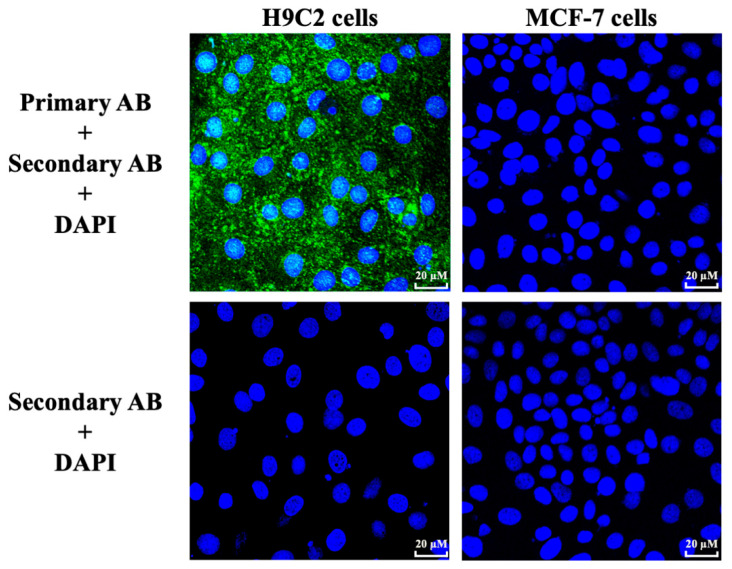
Indirect immunofluorescence staining of the GPR91 receptor on H9C2 and MCF-7 cells. Cells were labeled with 4′,6-diamidino-2-phénylindole (DAPI) and rabbit anti-GPR91 antibody (AB) revelated with fluorescent Alexa Fluor 488 secondary AB. A control without primary AB was made for both cell lines.
